# Bi-Directional Learning: Identifying Contaminants on the Yurok Indian Reservation

**DOI:** 10.3390/ijerph16193513

**Published:** 2019-09-20

**Authors:** Beth Rose Middleton, Sabine Talaugon, Thomas M. Young, Luann Wong, Suzanne Fluharty, Kaitlin Reed, Christine Cosby, Richard Myers

**Affiliations:** 1 Department of Native American Studies, University of California, Davis, CA 95616, USA; nstalaugon@ucdavis.edu; 2 Department of Civil and Environmental Engineering, University of California, Davis, CA 95616, USA; tyoung@ucdavis.edu (T.M.Y.); lhua@ucdavis.edu (L.W.); 3 Yurok Tribe Environmental Program, Klamath, CA 95548, USA; sfluharty@yuroktribe.nsn.us (S.F.); ccosby@yuroktribe.nsn.us (C.C.);; 4 Native American Studies, Humboldt State University, CA 95521, USA

**Keywords:** Indigenous, contamination, community, bi-directional, health

## Abstract

The Yurok Tribe partnered with the University of California Davis (UC Davis) Superfund Research Program to identify and address contaminants in the Klamath watershed that may be impairing human and ecosystem health. We draw on a community-based participatory research approach that begins with community concerns, includes shared duties across the research process, and collaborative interpretation of results. A primary challenge facing University and Tribal researchers on this project is the complexity of the relationship(s) between the identity and concentrations of contaminants and the diversity of illnesses plaguing community members. The framework of bi-directional learning includes Yurok-led river sampling, Yurok traditional ecological knowledge, University lab analysis, and collaborative interpretation of results. Yurok staff and community members share their unique exposure pathways, their knowledge of the landscape, their past scientific studies, and the history of landscape management, and University researchers use both specific and broad scope chemical screening techniques to attempt to identify contaminants and their sources. Both university and tribal knowledge are crucial to understanding the relationship between human and environmental health. This paper examines University and Tribal researchers’ shared learning, progress, and challenges at the end of the second year of a five-year Superfund Research Program (SRP) grant to identify and remediate toxins in the lower Klamath River watershed. Our water quality research is framed within a larger question of how to best build university–Tribal collaboration to address contamination and associated human health impacts.

## 1. Introduction

### 1.1. History and Landscape of Contamination at Yurok Indian Reservation (YIR)

**Figure 1:**
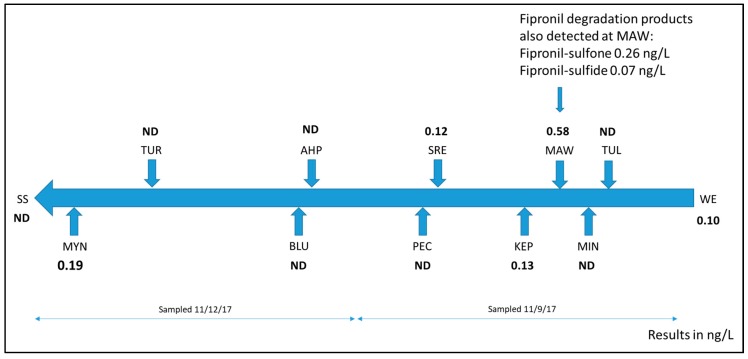
Fipronil and fipronil degradation product detections (ng/L) in the Klamath River and selected tributaries during November 2017.

The Yurok Tribe is currently the largest federally recognized Native American Tribe in the state of California, with over 6000 members. Since time immemorial, Yurok People have inhabited lands of the Lower Klamath and Trinity Rivers as well as along the Pacific Coast, extending from the mouth of the Little River near McKinleyville, California (CA), north approximately 70 miles to the mouth of Damnation Creek near Crescent City, CA. Yurok ancestral lands encompass an area of approximately 360,000 acres. The natural resources of the Klamath River, its surrounding lands, and the Pacific Ocean are central to the lives of Yurok People. Yurok People maintain cultural, economic, and spiritual ties to these ancestral lands through subsistence use and stewardship of traditional resources. The mosaic of marine, coastal, riverine, estuarine, lagoon, forestlands (redwood, fir, oak, cedar, spruce, and pine), prairielands, and high mountain environments is the cultural landscape of the Yurok, and Tribal members assert their “...traditional responsibility and aboriginal right to manage and utilize these places and resources, which has never been relinquished” [1]. Traditional Yurok law is woven into the Constitution, which mandates the Council to “…provide for the health, education, economy, and social wellbeing of our members and future members; restore, enhance, and manage the Tribal fishery, Tribal water rights, Tribal forests, and all other natural resources...” [2]. A portion of Yurok traditional lands are recognized as the Yurok Indian Reservation (YIR). The YIR is located in northwestern Humboldt and southwestern Del Norte Counties. The YIR consists of an approximately 59,000-acre corridor of land including the Klamath River, and extends for one mile from each side of the Klamath River, 46 miles from the mouth of the Klamath River to the Pacific Ocean upriver to approximately three miles above the town of Weitchpec (Figure 1).

The segment of the Klamath River running through Tribal lands is approximately 16% of the total length of the Klamath River measured from the outlet of Upper Klamath Lake to the Pacific Ocean. The Reservation includes two separate distinct populated areas, generally known as the Lower Reservation (area that surrounds the “lower” part of the Klamath River where it flows into the Pacific Ocean near Requa) and the Upper Reservation (area that surrounds the “up-river” portion of the Klamath River where the Trinity River flows into the Klamath River near Weitchpec).

Approximately 5090 acres are held in trust status within the YIR. The remaining lands in the YIR are fee lands, a majority of which are owned by Green Diamond Resource Company (GD, Seattle, WA, USA), and managed for industrial timber products. A small portion of the YIR consists of public lands managed by Redwood National/State Parks (RNSP), the United States Forest Service (USFS), and a number of other private landholdings. Approximately 960 people live on the YIR with most of the remaining Tribal members living nearby in Humboldt and Del Norte counties. The distinctive location of the Yurok lands that lie in the last 45 miles before the Klamath River enters the Pacific Ocean makes them a final catchment for the many residues used throughout the entire 15,751 square mile Klamath River watershed [3]. Increases in the adverse health conditions of Tribal members along with declines in the Klamath River fisheries over the last three decades have intensified interest and concern among Tribal members about the general condition of the Klamath River and their ancestral territories. As one Yurok elder, and member of the Superfund Community Advisory Committee stated, “Why, when we are trying to live a good life, do we end up with leukemia and fish and game contamination?” The Yurok Tribal Council and membership have identified the health of the Klamath River, its fishery, and the continued dependence on key subsistence species (including salmon, sturgeon, and pacific lamprey) as a primary concern for the Tribe and its future [4,5].

### 1.2. Yurok Tribe Environmental Program (YTEP) Work to Analyze, Remediate, Protect Community

The health of the environment, particularly the reservation environment and those traditional resources used for cultural, ceremonial, economic, and subsistence practices, are of primary importance to Yurok People. In 1998, the Yurok Tribe Environmental Program (YTEP) was formed in response to community-based concerns and continues to be led by community-based participatory research (CBPR) to identify and respond to community perceptions, priorities, and research objectives as approved by the Yurok Tribal Council. The YTEP’s mission is to protect the lands, air and water resources of the Yurok Reservation for the benefit of current and future generations of Tribal members. YTEP staff utilize science, traditional knowledge and environmental regulation for the purposes of enhancing Tribal sovereignty and expanding environmental regulatory authority of the Tribe to promote and protect these resources within the Yurok Reservation. The YTEP consists of three divisions: water, pollution prevention, and community and ecosystems.

Over the past 21 years, the YTEP has progressively developed extensive experience in surface and groundwater monitoring activities and expanded its capacity to monitor for environmental and public health concerns with awards from the U.S. Environmental Protection Agency (US EPA) under the Clean Water Act Section 106, 104 (b) (3) and 319 grants, General Assistance Program, and their National Center for Environmental Research [6,7]. In addition, the YTEP has received the US EPA’s Environmental Achievement Award due its innovation in environmental protection practices and ability to produce measurable results [8].

The YTEP has been successfully participating in and conducting environmental assessments, sampling, and research since its inception. The YTEP currently conducts water quality monitoring as a member of the Klamath Basin Monitoring Program, a workgroup with members from the U.S. Bureau of Reclamation, PacifiCorp, U.S. Fish and Wildlife Service, US EPA, State Water Resource Control Board, North Coast Water Quality Control Board, U.S. Forest Service, California Department of Water Resources, U.S. Bureau of Land Management, Salmon River Restoration Council, Karuk Tribe, Hoopa Tribe, and numerous other partners. Among other agencies, the YTEP also works collaboratively with the California North Coast Regional Water Quality Control Board for data collection needed to develop Total Maximum Daily Load (TMDL) discharge limits as well as the California Department of Public Health for monitoring of shellfish poisons within their Ancestral Territory.

The YTEP employs seven full-time staff with Bachelor’s degrees or higher and generally five technicians or interns with some college experience. All have been trained in rigorous quality assurance and control procedures and follow a US EPA-approved Quality Assurance Program Plan and numerous associated Sampling and Analysis Plans. Staff expertise includes the use and maintenance of data sondes, temperature probes, flow meters, data loggers, passive sampling, biota testing, and air quality monitoring devices. In addition, the YTEP performs robust data management and analysis, including entry into the Yurok Environmental Data Storage System (YEDSS) and transmission to the US EPA’s database for Air and Water Quality (AQS and WQX), and web published the YTEP Annual Water Year Report for water years 2005 through 2015.

With funding from sources including the Administration for Native Americans (2008–2009), and the Environmental Protection Agency’s National Center for Environmental Research: Science and Technology to Achieve Results program (2008–2012), the YTEP has been investigating the presence of selected contaminants and toxins in the local environment that are suspected to be associated with observed adverse health outcomes.

Under the 2008–2009 Administration for Native Americans (ANA) grant, the YTEP compiled data and information on potential and known point source locations of contaminants and toxins, identifying and verifying over 200 potential environmental pollutant sites, including illegal dumpsites, abandoned autos, herbicide spraying, burned homes, faulty septic systems, old mill sites, old mining sites, previously cleaned dumpsites, old home sites, and historic logging activity where machinery fluids were often improperly disposed into soil and waterways [9,10]. This data was stored in the YTEP’s secure and searchable Yurok Environmental Data Storage System (YEDSS) database. The YTEP has also completed numerous other recent environmental studies, including an Environmental Community Health Profile 2004–2011 [3], tissue sampling of subsistence food species for contaminants 2010–2012 [7] and a Source Water Assessment and Protection Program for Tribal Drinking Water Systems 2013–2014 [11]. An overview of the documented chemicals can be found in Table 1.

However, according to the YTEP’s 2010 final report on the ANA grant, there remains “insufficient and incomplete data on the extent of environmental contaminants throughout the YIR.” This is in conjunction with a jurisdictional framework that does not recognize the Yurok Tribe’s regulatory authority over all lands and waters within the boundaries of the Yurok reservation. This condition dates back to a history of appropriation of Yurok ancestral lands by the federal government, private companies, and private citizens [12,13,14]. The management of private lands in the basin, including privately owned land within the YIR boundaries, has been dominated by intensive timber harvest for the last 100 years. This harvest has included the use of pesticides that are currently banned on lands under Yurok Tribal jurisdiction [4,15]. This has impacted the Tribe’s federal trust natural resources [9,10]. Over the last several years, the Tribe has worked diligently, expanding programs and projects to increase information and capacity to identify and respond to threats to environmental and public health within reservation boundaries [10].

In addition to addressing intensive timberland management (including chemical spraying) on private lands within reservation boundaries, the Yurok Tribe has determined that there is significant impact to soil and surface water from illegal marijuana cultivation to the land and water on the Yurok Reservation. Possible impacts identified in a 2014 contracted study with Freshwater Environmental Services include: the presence of petroleum compounds used for generators, construction equipment, pumps, and other uses; the use of insecticides for insect control; the use of herbicides for vegetation control; the use of anticoagulant rodenticides for rodent control; and the use of fertilizers and nutrients to enhance growth [16].

On 5 September 2013, the Yurok Tribe passed the Yurok Tribe Controlled Substances Ordinance, explicitly prohibiting marijuana cultivation within Yurok Tribal boundaries. The Yurok Tribe selected the ordinance for ‘Emergency Adoption,’ because, as it states, “the Reservation community is under siege from unlawful cultivation of marijuana and drug manufacture” [15]. The legislation highlights the negative effects that marijuana cultivation has had on Tribal lands and waters, as well as on the social fabric of the community. In July 2014, the Yurok Tribe conducted a raid on illegal marijuana grows, dubbed ‘Operation Yurok.’ Although there were over 150 documented sites of marijuana cultivation prior to the raids, ‘Operation Yurok’ was only able to hit 69 of the sites [17]. The process of collaboration with local, state, and federal government entities included a morning briefing before crews of three would target their assigned locations. Marijuana cultivation brings with it the threat of violence and physical intimidation, which in a sense, holds Yurok People hostage within the boundaries of their own reservation. The National Guard and Humboldt County Sheriff would secure areas and then the Yurok Tribe was allowed to come in and take written and photographic documentation of the environmental damages [17]. Environmental damages included severe water diversions and impoundment, unpermitted clear cutting, human waste, and herbicides, pesticides, insecticides, and petroleum products [17]. The consequences and negative implications of marijuana cultivation on reservation land are many. Environmental contamination represents far more than simply resource scarcity; it impacts the very environment that makes Yurok People who they are.

### 1.3. Symptoms of Contamination (Human Health and Fisheries)

From 2008 through 2012, the Yurok Tribe Environmental Program conducted a project under a grant from the U.S. Environmental Protection Agency National Center for Environmental Research Grant, “Understanding the Cumulative Affects of Environmental and Psycho-Social Stressors that Threaten the Pohlik-lah and Ner-er-ner Lifeway: The Yurok Tribe’s Approach” [6]. The toxins and contaminants of interest and their related adverse health outcomes are can be viewed in Table 1.

As part of this project, the Yurok Tribe worked with the California Tribal Epidemiology Center (CTEC) to analyze the health outcomes of Yurok Tribal Members who visited United Indian Health Services at least once during the period between 2004–2009. His project represents 98% of the local bi-county, Yurok population and gives a comprehensive and representational snapshot of the local Yurok Tribal Members’ health at the community level [3]. The CTEC analysis shows that Yurok Tribal Members may have higher rates of cancer and proteinuria compared to national rates. The rate of cancer among the population studied is 683.5 per 100,000 people, compared to 190.4 per 100,000 American Indian/Alaska Native people nationally, and 484.0 per 100,000 for all races combined. The prevalence of proteinuria among the studied population is 2.6 percent, compared to the national prevalence of 1.1 percent. Proteinuria is a precursor of kidney disease although the rate of kidney disease among the Yurok Tribal Members is lower than the national average.

This study also examined perinatal outcomes, which the Superfund Community Advisory Committee has also cited as a concern. The study found that of the 189 pregnancies from 244 Yurok women seen at United Indian Health Services (UIHS, Arcata, CA), 27 of the pregnancies resulted in spontaneous abortion or fetal loss. This translates to a rate of 78.9 per 1000 pregnancies, or 9.3 percent of pregnancies. Further, 24 of the pregnancies resulted in a fetal malformation or infant anomaly. Because there is no consensus on diagnosis coding for spontaneous abortion, miscarriage, or stillbirth, comparisons with national data are impossible due to data inconsistencies. The CTEC analysis could not make any deterministic links between the known contaminants in the Klamath River environment and these health outcomes, but the documented adverse diagnoses demonstrate the need for further studies of local Yurok Tribal members’ health, links to local environmental contaminants, and the potentially disparate burden of disease and ill health within the Yurok Community living in Del Norte and Humboldt Counties.

Also as part of the US EPA project, in 2010–2012, the YTEP conducted, sampled and tested selected aquatic species for a range of contaminants that could impact resource and human health. The YTEP tested subsistence species and Klamath River water for more than 220 toxins and metabolites from 12 broad families/categories, returning positive results for biotoxins, dioxins, organochlorines, polycyclic aromatic hydrocarbons (PAHs), Polybrominated diphenyl ethers (PBDEs), polychlorinated biphenyls (PCBs), pyrethroids, and trace metals. These add to the cumulative impacts to Tribal Members through the ingestion exposure route. In general, detections were low with exceptions to five contaminant groups that exceed current public health or water quality criteria limits, including microcystins in Fall flows of Klamath River water and freshwater mussels, total PAHs in four species (Fall run Chinook salmon, Coho, lamprey, and razor clams), PCBs in whale blubber (comparing it to U.S. Food and Drug Administration (FDA) levels in red meat), pesticide residue in whale blubber (comparing it to FDA levels in red meat), and select trace metals (aluminum in Klamath River Spring flows and manganese in fresh water mussels) [7].

The YTEP has found that regional impacts from anthropogenic activities have impaired the Klamath River by way of both temperature and nutrients, resulting in the subsequent production of blue-green algae’s toxin microcystin. Routine surface water monitoring data document detections of microcystins on average seven weeks each year, from 2009 through 2013, exceeded levels above which adverse health effects could occur through incidental recreational exposure levels set by California Environmental Protection Agency, California State Water Resources Control Board (SWRCB), and California’s Office of Environmental Health and Hazard Assessment (OEHHA). In addition to the risk of microcystin exposure in the Klamath River surface waters, the YTEP’s subsistence species tissue testing found that freshwater mussels had detections of 64.2 ng/g of microcystin-LA [7]. This has the potential to add to the cumulative total toxic burden of microcystins that Tribal members may experience.

Table 1 provides a list of contaminants present in the Yurok environment, including in culturally significant species, and their impacts on human health.

Data gathered in the US EPA project document the complete exposure pathways of multiple environmental toxins and contaminants and indicate that exposure to the contaminants has occurred in the past, is presently occurring, or will likely occur in the future as contributors and sources of risk to the general Yurok Tribal Membership. Although the true or actual risk is unknown and the identification of a completed exposure pathway does not immediately imply health effects will occur, the project provided evidence that environmental contaminants and toxins are likely contributing to documented adverse health effects among Yurok Tribal Membership. While the direct correlation between adverse health effects and contaminants requires more research, the decline in salmon and other subsistence fisheries associated with the Klamath Hydroelectric Project and the contamination of subsistence fish species has decreased the availability of traditional foods, particularly salmon, and contributed to an increase in diabetes and heart disease rates [18].

### 1.4. Unique Exposure Pathways

All people living, working on, visiting, or otherwise present on the Reservation may be acutely exposed to hazardous substances at known or potentially impacted sites within the Reservation [19]. At these sites, they may come into contact with contaminated biota, soil, sediments, water, and/or vapors. The potential human exposure scenarios (i.e., exposure assessments) range from direct exposure to contaminants to casual indirect exposure through the uptake and bio-assimilation of contaminants in local waters, food sources, and culturally important plants that are ingested, inhaled, or handled. Examples of such sites include forestry and agriculture plots (including illegal marijuana grows) with pesticide, herbicide, fungicide, and rodenticide residues; timber and plywood mills with associated natural and industrial chemicals; fire suppression zones; active and legacy mines; and high-risk areas from activities such as the burning of wood products waste and plastics, methamphetamine production, and illegal dumping. Furthermore, Highway 101—which runs through the Reservation—gathers into one transportation corridor private and commercial vehicles that produce vapor and particulate emissions from gasoline and diesel fuel and create the potential for accidental spills into ground and surface waters on the Reservation.

Yurok Tribal members may face additional exposures and adverse impacts (beyond those among the general population) through Tribal lifeways that include cultural and ceremonial activities as well as subsistence fishing, hunting, and gathering [19]. Tribal members are integrally related to the environment in ways not typically accounted for in most exposure evaluation models, which reflect exposures largely received in urban and suburban settings and do not consider the extent of Tribal environmental contact [20,21,22]. The Yurok Tribe has resided along the Lower Klamath River, its tributaries, and surrounding lands, including the Pacific Coast, since time immemorial. Consequently, Tribal members have historically faced and continue to face psycho-social impacts from the contamination of sacred sites and natural resources, the loss of purity in traditional medicines and basketry species, the alteration of the cultural landscape, and the decline and loss of traditionally significant species. These injuries, coupled with the Tribe’s strong commercial and subsistence fisheries, high utilization due to economic reliance on other coastal and riverine resources, and extensive cultural programs and ceremonial activities, place the Yurok at severe risk of cumulative exposures from multiple contaminants.

Therefore, while Tribal members face the same routine exposures as do members of mainstream American communities to industrial additives and contaminants in commercial products and foods, exposures to such contaminants may be increased through Tribal-specific activities [19,20,21,22]. The Yurok population includes the same standard epidemiological sub-populations of physical vulnerability: children, pregnant women, and the elderly. Exposure across these sub-populations, however, is increased through routine subsistence activities and consumption. Differentiated sub-populations of cultural and ceremonial practitioners are at still higher risk due to additional completed exposure pathways such as chronic, frequent, and extended exposures to environmental pollutants via ingestion, inhalation, or dermal absorption. The YTEP has documented the specific exposure risks of Yurok Tribal members in scenario charts in the following four categories: food and drink (consumptive) pathways; subsistence pathways; cultural pathways; and ceremonial pathways. These charts can be found in the Yurok Tribe Interim Cleanup Standards for Contaminated Properties, adopted by Yurok Tribal Council in 2015. Taken together, these charts describe differentially exposed sub-populations; potentially complete pathways of exposure specific to Tribal members’ activities; and a range of possible contaminant transport mechanisms from suspect media: air, soil, soil vapor, sediments, biota, and ground and surface waters.

## 2. Materials and Methods: Working to Respond to Community Concerns

The YTEP employs a CBPR model in all work and research conducted by the department in environmental protection, restoration and management. The research questions and study objectives are developed in partnership with the community through scoping and consultation meetings with Yurok Natural Resources and Culture Committees, Yurok Tribal Council, and Tribal members at Council District Meetings. Further, the YTEP actively incorporates Yurok Traditional Ecological Knowledge (TEK) into all CBPR and planning activities. TEK informs resource protection and management priorities and strategies in all aspects of Yurok natural and cultural resources management. While environmental staff will not specifically collect additional TEK through ethnographic methods for this Project, TEK is relied upon to inform the development of environmental research—in this case, sampling methodology. TEK is the basis for Tribal members to evaluate their observations about what is outside “normal” and is relied upon for identifying priority issues, resources, and locations for monitoring and assessment with the goal of ultimately developing community-based, culturally appropriate strategies for protecting the human, community and ecological health.

University-based Superfund researchers are working to respond to Yurok Tribal members’ concerns that exposure to environmental contaminants (both historical and contemporaneous) is contributing significantly to disproportionate negative health outcomes within the community. As community members wrestle with current, unexplained health issues, they often point to the undocumented impacts of widespread aerial application of herbicides within the Klamath River watershed as part of timber management operations. Community members believe that direct exposure to these materials (e.g., through spray drift or broad application practices) or indirect exposure (e.g., via consumption of or contact with contaminated water, fish or plant materials) caused or contributed to the development of cancer and other diseases among residents of the Reservation.

A variety of challenges arise in the effort to provide a satisfactory resolution to these valid community concerns [23]. First, and probably most significantly, monitoring data from the period of presumed exposure (generally the 1980s and 1990s) is not sufficient to estimate the overall magnitude or temporal pattern of community exposures to the herbicide active ingredients. Even if data were available to estimate historical exposures accurately, a second level of challenges relate to gaps in scientific knowledge that limit the ability to establish links between exposures and negative health outcomes. Examples of such gaps include the impact of “inactive ingredients” such as solvents and surfactants, the synergistic effects of exposure to multiple chemicals in combination, and the possibility that treatment of herbicide-contaminated raw water supplies might produce disinfection byproducts of concern [24].

Another significant challenge inherent in the design of the Superfund Research Program is that it connects cutting edge biomedical and engineering research with translation of research results to community members concerned about past or current exposures to toxic substances. There is a fraught interface between research, which by its nature involves rapidly evolving methods with high dimensionality of information content, with efforts to provide a community with clear, accurate and actionable information. Within the UC Davis Superfund Research Center, there are a number of research project/community engagement interfaces but for this article, we will illustrate these challenges with a specific example: the effort to characterize the contaminants present in the Klamath River and its tributaries, an environment central to Tribal lifeways that is threatened by both industrial scale timber production and illegal marijuana cultivation in the upslope portions of the watershed. The characterization relies on recently developed methods that integrate gas and liquid chromatography using multiple ionization techniques to maximize the coverage of chemical analytes, particularly pesticides and their transformation products. The development, validation and application of these methods are described in detail in Moschet et al. [25]. Briefly, the water analyses are conducted by filtering a 1 L water sample through a glass fiber filter and then through a solid phase extraction cartridge to concentrate organic compounds. Compounds are recovered from the cartridge by eluting with organic solvents to prepare two extracts, one amenable to analysis via gas chromatography (ethyl acetate) and the other amenable to analysis via liquid chromatography (methanol). Analysis is conducted using liquid chromatography quadrupole time-of-flight mass spectrometry (Model 6530 LC-Q/TOF-MS, Agilent Technologies, Inc., Santa Clara, CA, USA) in both electrospray positive and negative ionization modes and gas chromatography quadrupole time-of-flight mass spectrometry (Model 7200B GC-Q/TOF-MS, Agilent Technologies, Inc., Santa Clara, CA, USA) in both electron ionization and negative chemical ionization modes. The resulting high-resolution mass spectrometric data (four instrument runs for each sample) is then analyzed for target compounds (primarily current use pesticides) and is subjected to suspect screening using high-resolution mass spectrometric databases (Agilent Pesticides, Water Contaminants and Toxicology Personal Compound Database Libraries (PCDLs) for liquid chromatography data, Agilent Retention Time Locked Pesticide PCDL and NIST17 library for gas chromatography data). The data will also be screened for non-target compounds (detected ion groups that are not identified via suspect screening) that are correlated with in vitro bioassay results during subsequent project phases.

## 3. Results

In the first phase of the UC Davis-YTEP collaboration, a primary goal was to broadly characterize the environmental quality to complement existing knowledge about contaminants surveyed in previous research efforts. In California’s Mediterranean climate, the first rains of the fall often carry a significant “first flush” of chemicals like herbicides and insecticides applied during the growing season into surface water bodies. Consequently, the initial water sampling was focused on sampling soon after early season rainfall in 2017, on 9 November (upriver sites) and 12 November (downriver sites). Twelve sites were sampled including an upriver Klamath River main stem site (Weitchpec), a downriver Klamath River main stem site (Wehl-kwew) and ten important tributaries with varied watershed land uses (Table 1). Five of the 21 target compounds were detected using GC-Q/TOF-MS (Table 2) and none of the 27 target compounds were detected by LC-Q/TOF-MS (Table 3). These target compounds, especially the pyrethroid insecticides, are widely detected in other California watersheds [25]. A depiction of the spatial distribution of the most widely detected target compound, fipronil, is shown in Figure 1. Fipronil is an insecticide widely used for pet flea control and other household uses; it is not registered for agricultural uses in California. Wastewater treatment plants have been identified as major sources to surface water in the San Francisco Bay area, with discharge concentrations of fipronil ranging from 13 to 88 ng/L and frequent detections of several degradates (fipronil sulfone and fipronil sulfide) at concentrations from 1 to 28 ng/L [26]. Fipronil and several degradates were detected in 100% of 53 samples collected across storm events in Cache Slough surface waters at concentrations from 0.1–13.3 ng/L [25].

**Figure 2:**
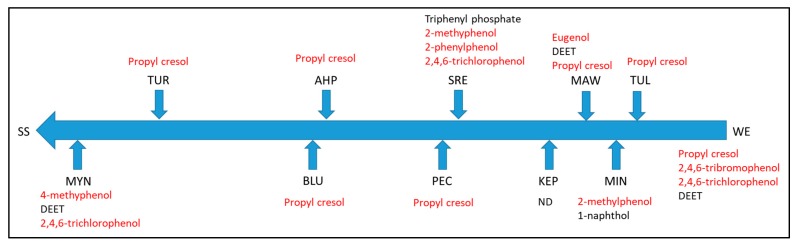
Suspect screening results for GC-Q/TOF-MS data.

**Figure 3:**
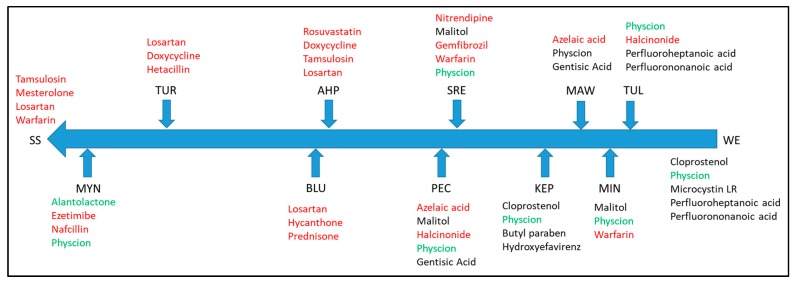
Suspect screening results for LC-Q/TOF-MS data.

Results of selected suspect compound screening results obtained from the GC-Q/TOF-MS data are summarized in Figure 2. Numerous phenolic compounds, both halogenated and non-halogenated, were detected and are indicated in red font in Figure 2. These compounds likely have diverse sources, including industrial activities, wood processing, consumer products and are also naturally occurring organic breakdown products. Other compounds detected include the insect repellent DEET and the widely used plasticizer and flame retardant triphenyl phosphate. Results from the target compound measurements using the LC-Q/TOF-MS are reported in Table 4, and indicate that none of the target pesticides or biocides were detected at levels above the analytical limit of quantitation (LOQ). Suspect compound screening of the same data against mass spectral databases identified numerous pharmaceutical compounds (indicated in red font in Figure 3) including gemfibrozil, losartan and warfarin (Figure 3). Some of the detected compounds are natural products (indicated in green font in Figure 3) that most likely arise from natural sources, including physcion (lichen pigment) and alantolactone. Other compounds detected (indicated in black font in Figure 3) include some perfluorinated heptanoic and nonanoic acids (degradates of compounds used as oil and stain repellents) and microcystin LR, an algal toxin that is known to be contributed to Yurok lands from algal blooms in upstream reservoirs. All of the detected compounds reported in Figure 2 and Figure 3 have not been confirmed by authentic pure compound standards but have sufficient similarity in their fragmentation patterns (electron ionization for GC or MS/MS fragmentation spectrum for LC) to be tentatively identified. Confirmation would require additional experimental effort. Impacts of most of these compounds are difficult to judge without quantification, but even with quantification the risks of many of these compounds would be difficult to determine based on existing knowledge. This situation illustrates the challenging clash between “academic or intellectual understanding and the lived experience of an issue,” as described by other researchers attempting to implement a community-based participatory research approach [27]. The gap between university researchers’ tools and community member experiences presents challenges in communicating results to Tribal members concerned with the quality of their environment and its potential impacts on their health and that of the organisms living in the Klamath River and tributaries.

This initial reconnaissance for contaminants led to discussions between Superfund Community Advisory Committee members and project researchers to plan ways forward based on these data. Plans were made to conduct time-resolved sampling at a subset of the initial sites to develop understanding of seasonal trends; to conduct in vitro bioassays to indicate whether samples contained compounds that activated or interfered with the action of the estrogen, androgen, thyroid or aryl hydrocarbon nuclear receptors; to better target suspected marijuana grow sites and former lumber processing facilities to characterize contaminant sources; and to better characterize sources, composition and fate of toxins that are produced by harmful algal blooms.

One unexpected opportunity that was identified in conversations between Tribal members on the Community Advisory Board and project researchers was the recognition that high-resolution mass spectrometric data collected as part of this project is unique in its ability to be queried in the future for compounds that may be believed to be benign based on present knowledge, but which are later found to be harmful. Advisory Board members saw the maintenance of this sort of data for future Tribal staff and members to retrospectively examine as part of their responsibility to be stewards of the environmental resources of their ancestral lands. Future activities will seek to build a data storage structure that supports and promotes such retrospective analyses of the data being collected now.

## 4. Discussion

### 4.1. The Role of Bi-Directional Learning in Evaluating Contamination in Indigenous Communities

A primary goal of the UC Davis Superfund Project is to implement bi-directional learning for university-based scientists and researchers and Tribal scientists and researchers. This approach recognizes the expertise of Tribal members and Tribal staff who have intimate, detailed, and long-term knowledge of the reservation environment, as well as the expertise of university-based researchers focused on particular contaminants and their specific pathways to impacting human health. The bi-directional approach acknowledges that Tribal members and academics have distinct and valuable knowledge bases and attempts to create opportunities to facilitate knowledge exchange. In addition to advancing knowledge on contaminants and their impacts on human health, the Superfund Research Project also focuses on practical outcomes of identifying environmental contaminants and mitigating their impacts on human health through the development of sensors to detect their presence, and remediation strategies to reduce their concentration.

As we enter the third year of our collaboration on the Superfund project “Biomarkers of Exposure to Hazardous Substances,” the Community Engagement Core (CEC) and the Superfund Deputy Director met with the Community Advisory Committee to reflect on both the successes and challenges of the UC Davis-Yurok Tribe collaboration thus far. The following section will highlight a series of challenges and our proposed, collaborative responses to them as we continue to work to improve the project and develop best practices for collaborative environmental health research.

### 4.2. Participation and Knowledge Exchange

The Superfund Research Program is composed of a large and diverse team of researchers with widely varying areas of focus. In Year 1, the Community Engagement Core worked with the Yurok Tribe Environmental Program (YTEP) to offer a series of trainings for university researchers on Yurok Tribal history, sovereignty, and the environmental research conducted by the YTEP. University researcher participation was generally low and, of those who attended the sessions, some applauded the content, but others wondered how it was relevant to their lab-based research. At the end of Year 2, the CEC offered a refresher training on Yurok sovereignty and environmental context in conjunction with a face-to-face dialogue between university researchers and YTEP staff on sampling plans and protocols. The more specific focus on research immediately following an introductory refresher presentation seemed to energize team members by making clearer links between research practices and community context.

### 4.3. Outreach and Dissemination of Information

The initial round of sampling took place in Year 1, and the CEC worked with Project 1 and the YTEP to develop factsheets summarizing the results to distribute at the annual Yurok Salmon Festival. The Festival is a lively, community-wide event featuring speakers, live music, traditionally cooked salmon, traditional sports, informational booths, and food, craft, and art vendors. Thousands of people attend, including many Tribal members who live both on and off of the Yurok reservation. In the initial project plan, CEC listed the Salmon Festival as the primary project outreach location. While the CEC booth featured a map of sampling locations as well as a chemistry demonstration by two University of California, Davis (UCD) researchers, in addition to summary factsheets documenting project activities and preliminary outcomes, meaningful outreach was hampered by the excitement of the Festival and other attractions. In response to this, UCD CEC and YTEP staff determined that, while CEC would still conduct similar outreach at the Festival next year, we will also plan 1–2 more focused community dialogues with specific groups, including weavers and possibly those suffering from illnesses that they believe to be related to environmental contamination. YTEP staff have also recommended that CEC and university researchers create a short video for the community documenting lab techniques. The latter will provide a visual summary of techniques being used to assess the safety of Klamath River water, thus increasing the range of media used to convey research processes and outcomes.

### 4.4. Communication of Realistic Project Outcomes

During the June 2018 project-wide site visit to Klamath, Superfund investigators were joined by UC Davis undergraduate students who were participating in the International Genetically Engineered Machine (iGEM) competition, in which students spend the summer working towards a proof-of-concept solution for a scientific problem, utilizing the tools of molecular biology. During the site visit, UC Davis iGem team learned about environmental pollution and adverse health outcomes at the Yurok Indian Reservation through presentations and conversations with Tribal Members and YTEP staff. Because the direct links between specific environmental contaminants and adverse health outcomes are still unclear, the team became interested in the possibility of leveraging natural protective stress–response pathways in mammalian cells by engineering them in ways that would turn on an easily measurable signal. The team also met with basketweavers and expressed their interest in applying this concept to the development of a sensor that the weavers could use to know the status of contaminants in the soil where they gather their weaving materials. After the site visit, the team explored the feasibility of building a cell-based tool that could be used to report a stress response in mammalian cells when these cells are exposed to chemicals of concern. Because they were only proving the concept, they did not use soil or water samples from the Yurok Indian Reservation, rather they generally tested the cell’s sensitivity to a variety of stressors. Their initial findings were encouraging, and they won multiple awards in the iGEM competition. Later, the YTEP communicated to the CEC that the basketweavers who met the iGEM team were under the impression the team would develop a sensor that they could use in the near future. The CEC met with the iGEM advisor to clarify the misunderstanding and asked the iGEM students to write a letter to the Yurok Tribal Members to explain that the iGEM team only had the time and resources to do a very preliminary part of the research to develop the sensor. The letter was distributed to the Community Advisory Committee.

The dynamics that occurred as a result of the inclusion of the UC Davis iGEM team in the site visit represent the type of miscommunication that can occur between research institutions and communities [28]. While bringing researchers and community members together can facilitate a fruitful exchange of information, it remains important that researchers communicate the scope of their work clearly. Specifically, this interaction highlights the importance of communicating realistic research and development timelines as well as the responsibility that researchers have in making efforts to ensure that community-engaged work is continued beyond short-term projects. The Superfund Research Project can learn from this interaction through consistent communication about research and development timelines and facilitating partnerships between the Tribe and other projects and programs at UC Davis that may help them in adjacent areas that may be out of the scope of the Superfund Research Program (SRP).

### 4.5. Responding to Community Concerns

As discussed in Section 4, one of the most significant challenges in our project thus far has been the disjunct between perceived impacts of environmental contamination and the initial sampling analysis results. While community members describe in impactful detail the range of cancers in particular that impact otherwise healthy community members who practice traditional subsistence lifeways, the data from the initial two rounds of sampling (in years 1 and 2) has not shown significant levels of contaminants. Researchers acknowledge both project-wide limitations of not being able to track the impact of historically applied chemicals, nor the cumulative impact of multiple chemical exposures, as well as the specific limitations of a small number of samples.

In order to begin to address the latter issue, the CEC Core leader organized a site visit between the Superfund Deputy Director (also a Project 1 researcher) and YTEP staff at the end of Year 2, which included driving throughout the Reservation to determine additional sampling locations, and to view specific legacy contaminant sites. The outcome of the meeting and the subsequent Advisory Committee meeting is a sampling plan that includes repeated sampling at specific high-priority locations, and sampling timed to catch specific, expected releases of chemicals into the watershed. The hope is that increased sampling and analysis data will paint a clearer and fuller picture of the contaminants present in the Klamath River watershed throughout the year, particularly during times when Tribal members have the most contact with the River and sediments.

In addition, university and Tribal researchers discussed the importance of comparing datasets of contamination and health issues over time, transferring sampling analysis data to the YTEP, and supporting the YTEP in advocacy for removal of aged lumber processing infrastructure left on the land, and the creation of Tribal lab facilities in which some of the techniques used at UC Davis for analysis and remediation of contaminants could be applied in Yurok facilities, on Yurok lands.

## 5. Conclusions

Yurok community members on the Community Advisory Committee, YTEP staff, and UC Davis researchers are building a cross-cultural, CBPR collaboration to address the health of Tribal members and their ancestral home in the Klamath River watershed. The work takes place in a painful context in which, as one Advisory Committee member explained, “The land was not only stolen; it was logged and poisoned.” Yurok Tribal members live with the effects of colonial policy towards their homeland and communities, including higher rates of cancers, miscarriages, learning disabilities, and other health impacts. In contrast, lab-based researchers are often very specifically focused on particular chemicals, techniques, or biological components. The Superfund project has provided an opportunity for researchers to think broadly about the need for their work to address current, dire situations in communities directly impacted by toxic legacies. Community members and YTEP staff are working to convey to researchers the importance of thinking multi-generationally: recognizing what elders lived through and working to clean the environment and leave a better legacy for generations to come. To improve community–university communication and knowledge exchange, the UC Davis Superfund Project has increasingly emphasized face-to-face dialogue (both on Tribal lands and in University facilities) between University and Tribal staff. This increased interaction has resulted in the direct assessment of challenges encountered in years 1 and 2, and specific plans to increase sampling and analysis and emphasize visual and dialogue-oriented conveyance of project outcomes in years 3 and 4, in order to work toward the successful development and dissemination of assessment and remediation tools by year 5.

## Figures and Tables

**Table 1:** Toxins present in Yurok culturally significant species and environment, and symptoms.

Toxins	Symptoms
(N-Methyl) Carbamates	overstimulation of nerves, causing muscle weakness dizziness central nervous system depression and pulmonary edema in serious cases, and headache salivation, sweating, nausea, vomiting, and abdominal pain diarrhea
Dioxins/Furans	skin damage neurological and immune system impairments in infants endocrine system disruption and associated insulin resistance, obesity, type II diabetes, and increased cancers of breast, testes, prostate and thyroid attention deficiencies lowered intelligence quotient (IQ)
Mercury	damage to the gastrointestinal tract and kidneys impaired neurological development and reduced peripheral vision, “pins and needles” sensations, and muscle weakness including impairment of speech, hearing and walking
Microcystins	rash hepatotoxicity with associated vomiting, diarrhea, abdominal pain, malaise, and muscle fasciculations abdominal breathing convulsions
Organochlorine Pesticides (1st group)	neurotoxicity and associated hyperexcitable state in the brain tremors seizures convulsions possible human carcinogen, with increased liver tumors and breast, testes, prostate and thyroid cancers immune disruption and associated non-Hodgkin lymphoma endocrine system disruption insulin resistance, obesity, and type II diabetes attention deficiencies lowered IQ
Organophosphate Pesticides (2nd group)	neurotoxicity and associated muscle weakness paralysis of the extremities headache muscle twitching seizures loss of consciousness, nausea and diarrhea respiratory depression and failure hypersecretion, with increased sweating, salivation, lacrimation and rhinorrhea increased incidence of Parkinson’s disease reduced levels of testosterone
Phenols including pentachlorophenol (PCP) and trichlorophenol (TCP)	thyroid endocrine system disruption, with associated insulin resistance, obesity and type II diabetes increased cancers, attention deficiencies, and lowered IQ

**Table 2:** Klamath River and tributary water sampling sites.

CK Mouth River Mile	Elevation	Watershed	Watershed Acres	Site Name	Description
43.5	60	Klamath	na	Weitchpec	Klamath River, mainstem at Weitchpec; used as baseline for water quality of the Klamath River entering the Reservation. River bank and river bar used for basketry materials; public boat ramp.
37.4	66	Tully	11,201	Tully Creek	Important cultural site for community/youth Culture Camps; Tan Oak gathering; willow harvesting; men’s sweats; and fish refugia. Has private domestic and drinking water intakes and 1 community system well/storage cistern under the direct influence of surface waters. Large watershed that is primarily managed for timber harvest including repeat herbicide application.
37.4	66	Tully	11,201	Homchow at Tully	Duplicate—Tully Creek.
35.9	109	Miners	2993	Miners Creek	Has multiple private domestic and drinking water intakes from surface water as well as springs in watershed. Upper reaches are extensively utilized for Cannabis growing operations. One of a series of critical fish rearing/refugia for Chinook and endangered Coho salmon.
34.5	360	Mahwah	1665	Mawah Creek	Has multiple private domestic and drinking water intakes from surface water as well as springs in watershed. Upper reaches are extensively utilized for Cannabis growing operations. One of a series of critical fish rearing/refugia for Chinook and endangered Coho salmon.
32.2	43	Kep’el	5396	Kep’el Creek	Traditional Salmon Dance/ceremony when fish dam was repaired, not currently practiced; take old hatchery road to community swimming hole/picnic area; important fish rearing area/cold water refugia and some spawning for first mile of creek. Main public water system uses this surface water; and scattered private systems from surface water and springs.
25.6	35	Sregon	38	Sregon	Ceremonial Dance grounds for one weekend each year for Brush Dance—primary participants (Medicine Boy, Medicine Girl, Medicine woman) fast and sweat 4–10 days; 200–300 participants camping, drinking water.
25.6	45	Sregon	38	Sregon Raak	Creek at Highway 169, Post Mile 15.6. Source is spring utilized for ceremonies and public traditional drinking source; along river, there are several important fish camp areas—5 fishing holes.
24.5	26	Pecwan	17,652	Pecwan Creek	Cultural significant site; location every two years for the Jump Dance, the ceremonial renewal dance. It is a ten-day event and a hand full of men who are separated from the rest of the people fast, sweat, and bathe in creek water. Also involved are Karuk, Hupa people; they come to help balance the world and these folks drink, swim, and bathe in this creek; there are hundreds of people during these ten days. Multiple small community and private source water of surface waters and springs. Fish rearing and cold water refugia for Chinook and endangered Coho salmon. Watershed managed primarily for timber with some active logging.
17.0	32	Ah Pah	10,307	Ah Pah Creek Mouth	Has multiple private domestic and drinking water intakes from springs in watershed. One of a series of critical fish rearing/refugia for Chinook and endangered Coho salmon. Traditional village site sits above the mouth of Ah Pah Creek; currently used as a ‘living-village’ for cultural workshops and working toward revitalizing cultural ceremonies/Jump Dance. Watershed predominantly private property of Green Diamond Resource Company and intensively managed for commercial timber with repeat pesticide applications, including 2016 and 2017.
17.0	37	Ah Pah	10,307	GD WQ station	Ah Pah Creek at the Green Diamond Resource Company’s water quality station on South Fork.
17.0	37	Ah Pah	10,307	Homchow at AhPah 2	Duplicate—Ah Pah.
16.1	11	Blue	80,167	Blue Creek	This is the largest tributary watershed in the Lower Klamath River Basin and extremely import to the Yurok Tribe as it is a major fish-bearing stream (chinook, coho, steelhead, cutthroat, sculpin, speckled dace, three spine stickleback, Klamath small-scale sucker, and lamprey eel) with a distinct genetic chinook salmon population. Recently acquired from the Green Diamond, who in the past managed it for commercial timber. Under Yurok ownership, it will be managed in traditional ways and as a salmon sanctuary into perpetuity. In addition, it contains much of the Yurok sacred “High Country”.
5.5	13	Turwar	20,345	Turwar Creek	Has a private public water system, multiple private domestic and drinking water intakes from springs and private wells in watershed; approx 50 homes. Important fish rearing/refugia and site of one of the first fish habitat restoration projects by Yurok. Site of historic Arrow Mills lumber mills. Lower drainage significantly altered by the construction of the Klamath River levee system in late 1960–1970s. Lower watershed mostly rural residential property but upper reaches of watershed managed for commercial timber production.
1.3	2	Mynot	14,996	Hunter-Mynot-Spruce	Has private public water system, multiple private domestic and drinking water intakes from springs and private wells in watershed. One of a series of critical rearing and juvenile over-wintering areas for the endangered Coho salmon.
0.2	6	Klamath	na	Wehl-kwew	Klamath mainstem in South Slough wetlands of estuary at Wehl-kwew. Ceremonial Dance Grounds at mouth of river within National Redwoods Park with lots of tourist visitors as well as Tribal member use. Fish move through slough and shelter and overwinter within the channel. It is brackish water.

**Table 3:** Target Compound Results via GC-Q/TOF-MS.

Detected	Detected < LOQ	Never Detected	
Chlorpyrifos	Chlorothalonil	Bioallethrin	Novaluron
Fipronil		Prallethrin	Fipronil-desulfinyl
Fipronil-sulfide		Tetramethrin	Fipronil-desulfinyl amide
Fipronil sulfone		Bifenthrin	Fipronil amide
		Phenothrin	Deltamethrin
		Cyhalothrin	Esfenvalerate
		Cyfluthrin	Cypermethrin
		Cyphenothrin	Permethrin

LOQ = Limit of Quantitation.

**Table 4:** Target Compounds Analyzed by LC-Q/TOF-MS; None were detected.

	Compounds	
Triclocarban	Triclosan	Clomazone
Imidacloprid	Methomyl	Chlorantraniprole
Simazine	Thiamethoxam	Azoxystrobin
Diuron	Dimethoate	Metolachlor
Propanil	Thiacloprid	Difenoconazole
2-phenylphenol	Hexazinone	Thiobencarb
Boscalid	Propoxur	Pyriproxyfen
Methoxyfenozide	Cyprodinil	Pendimethalin
2,4-dichlorophenoxyacetic acid (2,4-D)	2-Methyl-4-Chlorophenoxyacetic Acid (MCPA)	Diethyltoluamide (DEET)
